# Editorial: The Role of Natural Killer Cells in Autoimmune Diseases

**DOI:** 10.3389/fimmu.2021.786190

**Published:** 2021-11-01

**Authors:** Alessandra Fierabracci, Domenico V. Delfino, Mary A. Markiewicz, Antonio La Cava

**Affiliations:** ^1^ Infectivology and Clinical Trials Research Department, Bambino Gesù Children’s Hospital, Istituto di Ricovero e Cura a Carattere Scientifico (IRCCS), Rome, Italy; ^2^ Section of Pharmacology, Department of Medicine and Surgery, University of Perugia, Perugia, Italy; ^3^ Department of Microbiology, Molecular Genetics & Immunology, University of Kansas Medical Center, Kansas City, KS, United States; ^4^ Department of Medicine, University of California Los Angeles, Los Angeles, CA, United States; ^5^ Dipartimento di Biochimica e Biotecnologie Mediche, Federico II University of Napoli, Naples, Italy

**Keywords:** NK cells, autoimmune diseases, autoimmunity, immune regulation, immune system

## NK Cells and Autoimmune Disease

The innate immune system is a first line of host defense against infectious agents and prevents tumor development. It acts in concert with the adaptive immune system to maintain effective immune responses and to protect against immune-mediated diseases while avoiding self-tissue damage. However, an impairment and/or a dysregulation of the complementary activity of the two systems can unbalance immune homeostasis and lead to loss of immune self-tolerance, with aberrant immune responses and autoimmune disease. In this regard, although a contribution of the adaptive immune cells to mechanisms of immune tolerance and balance between effector and regulatory responses has long been object of investigations, an important role is also contributed by innate immune cells in the control of autoimmune diseases, either directly or indirectly. This collection focuses on the influence of innate NK cells in autoimmunity, in addition to the better known role of NK cells in the protection from virally-infected and transformed cells.

In a comprehensive overview, Kucuksezer et al. described the biology and functions of NK cells with a special focus on their up-to-date classification, development and maturation, transcriptional profile, and receptor expression. They discussed the delicate balance between activating and inhibitory Killer Ig-like receptors (KIR) and their ligands in NK cell homeostasis, together with the influences by genetic and environmental factors and how altered NK cell functions could play a role in the pathogenesis of several autoimmune and autoinflammatory disorders. The authors also highlighted the potential use of NK cells in a growing number of therapeutic interventions, especially in autoimmunity and cancer, acknowledging that more investigation is needed to validate hypotheses and to clearly establish a link between NK cells and underlying pathologies.

Liu et al. discussed the divergent roles NK cells in different autoimmune diseases, whereby these cells can either protect or promote disease acceleration depending on the subsets involved, the disease stage, and the microenvironment. The authors elaborated on how a better understanding of these aspects could benefit the design and use of NK cell-targeted therapies.

Gianchecchi et al. overviewed the main phenotypic and functional characteristics of NK cells and their role as potential therapeutic targets in autoimmune disorders. NK cell numbers are often numerically and/or functionally reduced in patients with autoimmune conditions including type 1 diabetes (T1D), rheumatoid arthritis (RA), systemic-onset juvenile rheumatoid arthritis, systemic lupus erythematosus (SLE), primary biliary cholangitis systemic sclerosis, Sjögren’s syndrome, inflammatory bowel disease, multiple sclerosis, and Behcet’s disease. The authors indicate that some molecules expressed by NK cells such as soluble CD83, the B isoform of chemokine-receptor 3 (CXCR3) and the checkpoint inhibitor T-cell immunoglobulin and ITIM domain (TIGIT) could be potential therapeutic targets due to their role in the pathogenesis of autoimmune diseases.

Gomez-Munôz et al. measured the frequency and total counts of NK cells in the peripheral blood of pediatric patients with T1D at different stages of the disease. NK cells were divided into four subsets by flow cytometry: 1) CD56^dim^CD16^+^ or effector NK cells (NK_eff_); 2) CD56^bright^CD16^−^ or regulatory NK cells (NK_reg_); 3) intermediate CD56^bright^CD16^+^ NK cells; and 4) CD56^dim^CD16^−^ NK cells. These subsets were analysed at disease onset, partial remission, 8 months (for non-remitters) and 12 months of disease progression. The authors found that both total NK cell numbers and all four subsets were impaired at early stages of T1D. Total NK and NK_eff_ percentages were reduced as compared to controls due to extravasation into pancreatic islets (this continued during disease follow-up). Instead, NK_reg_ increased during early disease stages. The reduction of intermediate NK and CD56^dim^CD16^-^ NK cells at partial remission stage suggested immunoregulatory attempts, while a reduction of CD16 expression in total NK and NK_eff_ cells one year after diagnosis was suggestive of cell exhaustion. The authors propose that these alterations of NK cell subsets during disease progression could represent potential disease biomarkers.

Horwitz et al. described a newly identified population of NK cells endowed with immunoregulatory properties in mice that developed a graft versus host disease (GVHD) which resembled SLE. The model employed was that of chronic GVHD that follows the transfer of donor CD4^+^ T cells from DBA/2 mice into (C57BL/6 × DBA/2)F_1_ (BDF1) mice. Treatment of those mice with tolerogenic polymer poly(lactic‐co‐glycolic acid) (PLGA) nanoparticles (NPs) targeted to CD2^+^ cells induced NK cells that critically contributed to the protection of the mice from disease progression *via* a TGF-β-dependent mechanism.

Hervier et al. reported that soluble MICA (sMICA) and MICB (sMICB) were present in sera of adult patients with SLE at a higher concentration than in healthy individuals, and that membrane-bound MICA was induced on monocytes by mononucleosomes (major lupus autoantigens). Co-culture of mononucleosome-activated PBMCs with NK cells led to NK cell activation that was abrogated with monocyte depletion. Interestingly, the activation of NK cells from SLE patients with monocyte-treated nucleosomes was less than that of NK cells from healthy individuals, perhaps due to chronic NKG2D stimulation or the presence *in vivo* of sMICA and sMICB. Together, these data indicated that mononucleosomes induction of MICA expression by monocytes from SLE patients could result in chronic NK cell activation and subsequent repression of NK cell function.

## Conclusions

This Research Topic provides updates on the influences of NK cells on the initiation, progression, and outcomes of autoimmune diseases. NK cells can behave dissimilarly in the development of different autoimmune disorders and during different disease stages, by either promoting or protecting the host. Although the modulation of NK cell activity has been proposed as a tool of management of autoimmune diseases, additional studies are needed to move the laboratory results to the patient’s bedside. Furthering our understanding of the mechanisms by which NK cells can influence autoimmune diseases could have relevant implications in the design of new strategies of NK cell-targeted therapeutic intervention.

## Author Contributions

All authors made a substantial, direct and intellectual contribution to the work, and approved it for publication.

## Funding

The work was supported by the Italian Ministry of Health Ricerca Corrente RC2021_INFETT_FIERABRACCI to AF.

## Conflict of Interest

The authors declare that the research was conducted in the absence of any commercial or financial relationships that could be construed as a potential conflict of interest.

## Publisher’s Note

All claims expressed in this article are solely those of the authors and do not necessarily represent those of their affiliated organizations, or those of the publisher, the editors and the reviewers. Any product that may be evaluated in this article, or claim that may be made by its manufacturer, is not guaranteed or endorsed by the publisher.

